# Exercise improves depression through positive modulation of brain-derived neurotrophic factor (BDNF). A review based on 100 manuscripts over 20 years

**DOI:** 10.3389/fphys.2023.1102526

**Published:** 2023-03-08

**Authors:** Monèm Jemni, Rashid Zaman, Frederick Robert Carrick, Neil David Clarke, Michel Marina, Lindsay Bottoms, Jagdeep Singh Matharoo, Roger Ramsbottom, Norman Hoffman, Shad James Groves, Yaodong Gu, Ferman Konukman

**Affiliations:** ^1^ Faculty of Physical Education, Ningbo University, Ningbo, Zhejiang, China; ^2^ The Carrick Institute of Neuroscience, Cape Canaveral, FL, United States; ^3^ Centre for Mental Health Research in association with The University of Cambridge, Cambridge, United Kingdom; ^4^ Department of Psychiatry, The University of Cambridge, Cambridge, United Kingdom; ^5^ University of Central Florida College of Medicine, Orlando, FL, United states; ^6^ MGH Institute of Health Professions, Boston, MA, United States; ^7^ Centre for Sport, Exercise and Life Sciences, Coventry University, Coventry, United Kingdom; ^8^ Institut Nacional d'Educació Física de Catalunya (INEFC), Sport Performance, Barcelona, Spain; ^9^ School of Life and Medical Sciences, University of Hertfordshire, Hatfield, Hertfordshire, United Kingdom; ^10^ The University of Greenwich, London, United Kingdom; ^11^ Sport and Health Sciences, Faculty of Health and Life Sciences, Oxford Brookes University, Oxford, United Kingdom; ^12^ Department of Physical Education, College of Education, Qatar University, Doha, Qatar

**Keywords:** BDNF expression, exercise mode, neuroplasticity, depression treatment, physical activity intervention

## Abstract

The aim of this review was to explore the relevant neurobiology and the association between peripheral levels of brain-derived neurotrophic factor (BDNF) and acute and short to long-term exercise regimes, as well as its relation to depression and antidepressant treatment. A 20-year literature search was conducted. The screening process resulted in 100 manuscripts. Antidepressants as well as acute exercise, particularly high-intensity, elevates BDNF in healthy humans and clinical populations, as evidenced from aerobic and resistance-based studies. Although exercise is increasingly recognised in the management of depression, acute and short-term exercise studies have failed to establish a relationship between the severity of depression and changes in peripheral BDNF. The latter rapidly returns to baseline, possibly indicating a quick re-uptake by the brain, aiding its neuroplasticity functions. The timescale of administration needed for the antidepressants to stimulate biochemical changes is longer than similar increases with acute exercise.

## 1 Introduction

Globally, more than 264 million people of all ages suffer from depression (James et al., 2018). Depression is different from the usual mood fluctuations and short-lived emotional responses to challenges in everyday life. When long-lasting and with moderate or severe intensity, depression may become a serious health condition (WHO, 2021). The total cost of depression in Europe is rising and was estimated to be €118 billion (Valladares et al., 2009). The COVID-19 pandemic has dramatically affected these figures. The number of adult people living with depression has more than doubled from November 2020 to early 2021 in the United Kingdom. Around one in five (21%) adults experienced some depression up to March 2021 (Puccinelli et al., 2021; Williams et al., 2021).

The United Kingdom National Health Service (NHS) has published a record numbers of people seeking help for mental healthcare (4.3 million people, including one million under 18 years of age) (Dubicka, 2021 - The Royal College of Psychiatrists, 2021). 1.68 million more mental health sessions were delivered during the pandemic compared to 2019 (Dubicka, 2021). These records have led the Royal College of Psychiatry to warn that the pandemic may result in a generation being lost to life-long illness (Hymas, 2021). The repeated lockdowns, the school closures, the empty colleges, and the cancellation of exams threaten to create a mental health crisis that could endanger the current generation of children and adults for years to come (Hymas, 2021). These concerns are amplified by acknowledging that antidepressants are no cure-all for patients’ mental health (Hymas, 2021). Garcia-Adasme et al. (2021) reported that 56.3% of children under the age of seven placed into confinement during the COVID-19 pandemic had four or more anxiety-related symptoms with the most frequent symptoms being tantrums, emotional changes, restlessness, and the fear of being alone. Expert politicians have referred to a “ticking time bomb” of mental health problems seeing the stress, anxiety, depression and many other mental issues that even healthcare professionals were experiencing during this pandemic. Mental health, has indeed become the number two priority worldwide after COVID-19 (WHO, 2021). The question that everyone is seeking to answer is, *“Are there any other ways to treat depression besides medication?”*. In this context, a randomized controlled trial demonstrated that both physical high-intensity interval training (HIIT) and moderate-intensity training (aerobic) effectively decreased anxiety, stress, and depression, as well as increased resilience during the COVID-19 confinement (Borrega-Mouquinho et al., 2021).

Proposed mechanisms implicated in the pathophysiology of depression include neural alterations in the brain (Castren and Rantamaki, 2010). These alterations are associated with biochemical changes in growth factors such as vascular endothelial growth factor (VEGF), insulin-like growth factor-1 (IGF-1) and brain-derived neurotrophic factor (BDNF). Alterations in BDNF activity results in aberrant cortical function, neuronal dysfunction, and psychiatric disorders (Markham et al., 2014). Of specific interest is BDNF which is a commonly studied dimeric polypeptide neurotrophic factor belonging to the neurotrophin family of growth factors (Hallbook, 1999) and is involved in crucial aspects of neuronal functioning (McAllister, 2000; Kovalchuk et al., 2002) including differentiation, axonal growth, neuronal survival, and synaptic plasticity (Huang et al., 2008; Markham et al., 2014). In fact, BDNF is expressed in all regions of the brain, although most abundantly in the hippocampus, amygdala, cerebral cortex, and cerebellum (Hofer et al., 1990). Furthermore, BDNF is produced within skeletal muscle during contraction, however, it appears that muscle-derived BDNF does not enter the bloodstream (Matthews et al., 2009). However, blood levels of BDNF have been found to strongly correlate with brain levels (Klein et al., 2011). In the blood, most BDNF is stored inside platelets with very little being freely available in the plasma (Fujimura et al., 2002). Therefore, measurements of peripheral BDNF must account for the source, whether plasma or serum, serum containing between 25 and 120 times the amount of BDNF with these numbers being greatly affected by the coagulation time and centrifugation strategy (Gejl et al., 2019). All of the above support the findings provided by Brunelli et al. (2012) that showed when correcting for the blood plasma loss, the observed increase of circulating BDNF after intense and/or moderate exercise would be less significant. All these pieces of evidence make it untenable to correlate or convert plasma levels to serum or *vice versa* when comparing studies that use differing methodologies. In fact, Gejl et al. (2019) suggest that plasma and serum BDNF pools are ‘independent measures of diverse biological relevance’.

Li et al. (2007) suggest that platelet activation and coagulation are induced by intense physical exercise and that BDNF is thus released from its storage form inside platelets into the plasma where it would be free to bind to available receptors. Since BDNF that is released peripherally freely crosses the blood-brain barrier, this growth factor would be available to neurons in the brain to impact plasticity (Klein et al., 2011). Further, exercise causes muscles to secrete myokines such as Irisin which also crosses the blood-brain barrier and evokes the release of BDNF by such areas as the hippocampus, impacting brain levels and blood levels (Jodeiri Farshbaf & Alviña, 2021).

There is strong evidence from experimental and post-mortem studies to demonstrate that serum BDNF levels are reduced in patients with clinical depression without medication (Chen et al., 2001; Shimizu et al., 2003; Karege et al., 2005; Piccinni et al., 2008a; Fornaro et al., 2015; Phillips, 2017; Sheldrick et al., 2017). It is so generally accepted that BDNF plays a major role in major depressive disorder (MDD) that BDNF-knockout mice are widely used to examine the experimental role of other markers, inflammatory responses, and gene expression in MDD animal models (Jodeiri Farshbaf & Alviña, 2021; Miyanishi & Nitta, 2021; Li et al., 2022).

Antidepressants appear to positively elevate concentrations of BDNF, as noted in clinical studies of individuals living with depression (Krishnan and Nestler, 2008; Sen et al., 2008). However, there are drawbacks associated with antidepressants including the growing costs of medication, negative side effects (Cascade et al., 2009), simultaneous use of multiple medicines for treatment-resistant depression (polypharmacy) (Dunner, 2014), non-responders to treatment (Schechter et al., 2005) and compliance issues amongst different age groups and ethnicities (Murphy et al., 2013). Exercise is as effective as antidepressants in treating depression (Blumenthal et al., 1999; Heyman, et al., 2012; Kandola et al., 2019). The effect of exercise on depression has been the subject of research for several decades (Scott and Dickey, 2003). Many studies and/or clinical trials have reported reductions in the severity of depression following exercise (Dimeo et al., 2001; Dunn et al., 2005; Singh et al., 2005; Kandola et al., 2019). Importantly, exercise has been associated with neuronal regeneration, protection, and adaptation (Castellano and White, 2008). Acute and longer-term exercises have elicited increases in peripheral and brain BDNF, with evidence from many studies (Tang et al., 2007; Tavares et al., 2007; Zoladz et al., 2008; Gustafsson et al., 2009; Rasmussen et al., 2009; Yarrow et al., 2010; Goekint et al., 2011) including a review (Knaepen et al., 2010). Previous literature on exercise and BDNF has largely neglected the role of exercise in the treatment of depression. Therefore, the present paper aims to review the relevant neurobiology and the relationship of peripheral BDNF levels to acute and short to long-term exercise programs, depression and antidepressant treatment.

## 2 Search strategy and selection criteria

**FIGURE 1:**
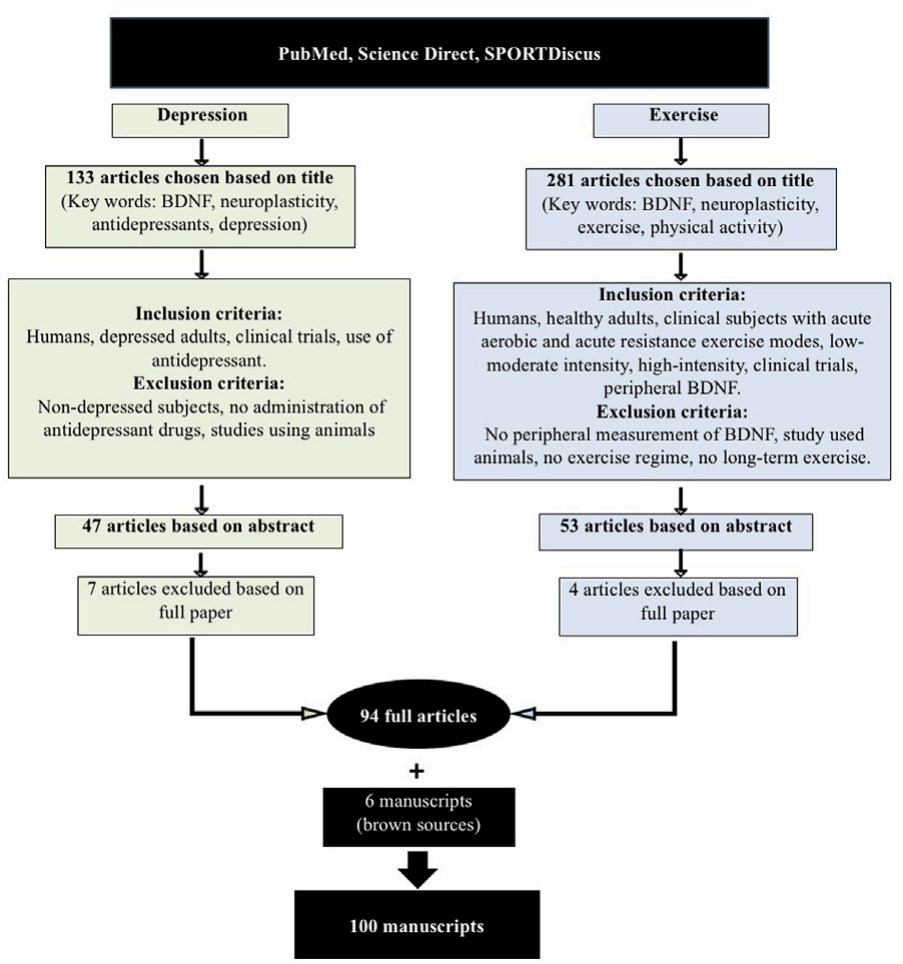
PRISMA flow diagram for search and selection process.

An extensive literature search was conducted as per PRISMA guidelines to include papers published between 2001 and May 2021, using the three databases: PubMed, Science Direct and SportDiscus^®^. Such an extensive period of time was retained to witness the evolution of the related-concepts throughout the years whilst providing an accurate evidence-based review outcome. The three databases were chosen for a few reasons, amongst them the fact they are free and would enable the readers to, at least, access the abstracts if not the full articles. All together, they cover multi-disciplinary, peer-reviewed journal articles in the fields of science, life and biomedical sciences, medicine, physical activity, and exercise related literature. The databases were screened using the keywords: brain derived neurotrophic factor and/or the acronym BDNF, neuroplasticity, exercise, physical activity, antidepressants, depression. The entire review protocol is shown in Figure 1.

A first screening process resulted in 133 articles that were related to depression and BDNF and a further 281 studies related to exercise and BDNF. Further inclusion criteria were applied to the 133 studies, as follows: humans, depressed adults, clinical trials, and the use of antidepressant medications by subjects. Exclusion criteria were: non-depressed subjects, no administration of antidepressant drugs to subjects, or studies using animals. These criteria narrowed down the number of papers to 47, based on their abstracts. Seven papers were further excluded after reading the full papers, which led to 40 depression and BDNF-related full papers being retained. The inclusion criteria applied to the 281 exercise and BDNF-related studies were as follows: humans, healthy adults and clinical subjects with acute aerobic and acute resistance exercise modes, low-moderate intensity, high-intensity, clinical trials and peripheral BDNF measurement. Studies were excluded if there was no peripheral measurement of BDNF, studies that used animals, no exercise regime used or if long-term exercise protocols were used. These criteria cut down the number of papers to 53, based on their abstracts. Only four articles were subsequently excluded after reading the full papers, leading to 49 exercise and BDNF-related full papers being retained. Five extra papers were recommended by the reviewers and added. A total of 94 full articles was therefore retained. Six further articles/reports from websites and brown sources were also retained. Therefore, the full review was undertaken on 100 articles.

## 3 Results and discussion

### 3.1 Exercise and depression

There is growing support for the role of exercise in the reduction of the severity of depression, as suggested by a number of studies and/or clinical trials demonstrating a reduction in depression severity (Dimeo et al., 2001; Dunn et al., 2005; Singh et al., 2005). Although the majority of studies have incorporated aerobic exercise regimes, studies have shown that resistance-based training is also effective in lowering depression (Singh et al., 2005). Table 1 provides specific definitions of terms that will be used throughout the paper.

**TABLE 1:** Specific terms’ definitions.

Term	Definition
Exercise (Oxford learners dictionary)	Physical or mental activity to undertake to stay healthy or become stronger. In this particular study, we refer to exercise that could light/gentle, moderate, strenuous/vigorous or high intensity
Physical activity (WHO)	WHO defines physical activity as any bodily movement produced by skeletal muscles that requires energy expenditure. These include movement during leisure time, to get to and from places, part of a job, sports, ect
Warm-up (Oxford learners dictionary)	A set of exercises done before a game, contest, or workout
Training (Oxford learners dictionary)	Training is teaching, or developing in oneself or others, any skills and knowledge or fitness that relate to specific competencies. It has specific goals of improving one’s capability, capacity, productivity and performance. In this particular study, we refer to physical training
Sport (Oxford learners dictionary)	Activity performed for pleasure and that needs physical effort or skill, usually performed in a special area and according to well defied rules
Aerobic exercise (Oxford learners dictionary)	Physical exercise especially designed to improve the function of the heart and lungs which could be undertaken in different forms (individual or group), settings (indoors, outdoors), and even with music
Endurance exercise/training (Oxford learners dictionary)	The ability to continue doing something painful or difficult for a long period of time without complaining. In this particular study, we refer to physical endurance, generally repeating an exercise/activity over a long period of time
Anaerobic exercise/training (study.com)	Activities that are generally short, quick and high-intensity, which push the body to perform without the use of oxygen
Resistance exercise/training (Cambridge dictionary)	Activity of lifting heavy objects for exercise, especially to improve the strength of the muscles. In this particular study, we refer to resistance as exercises/training that uses simple equipment, such as free weights, which can be moved by different forms of muscle contractions
HIIT (Oxford learners dictionary)	A form of exercise in which you do short periods of very hard physical activity with easier exercises in between
Acute exercise (Collins Dictionary)	Exercise of relatively short duration

**FIGURE 2:**
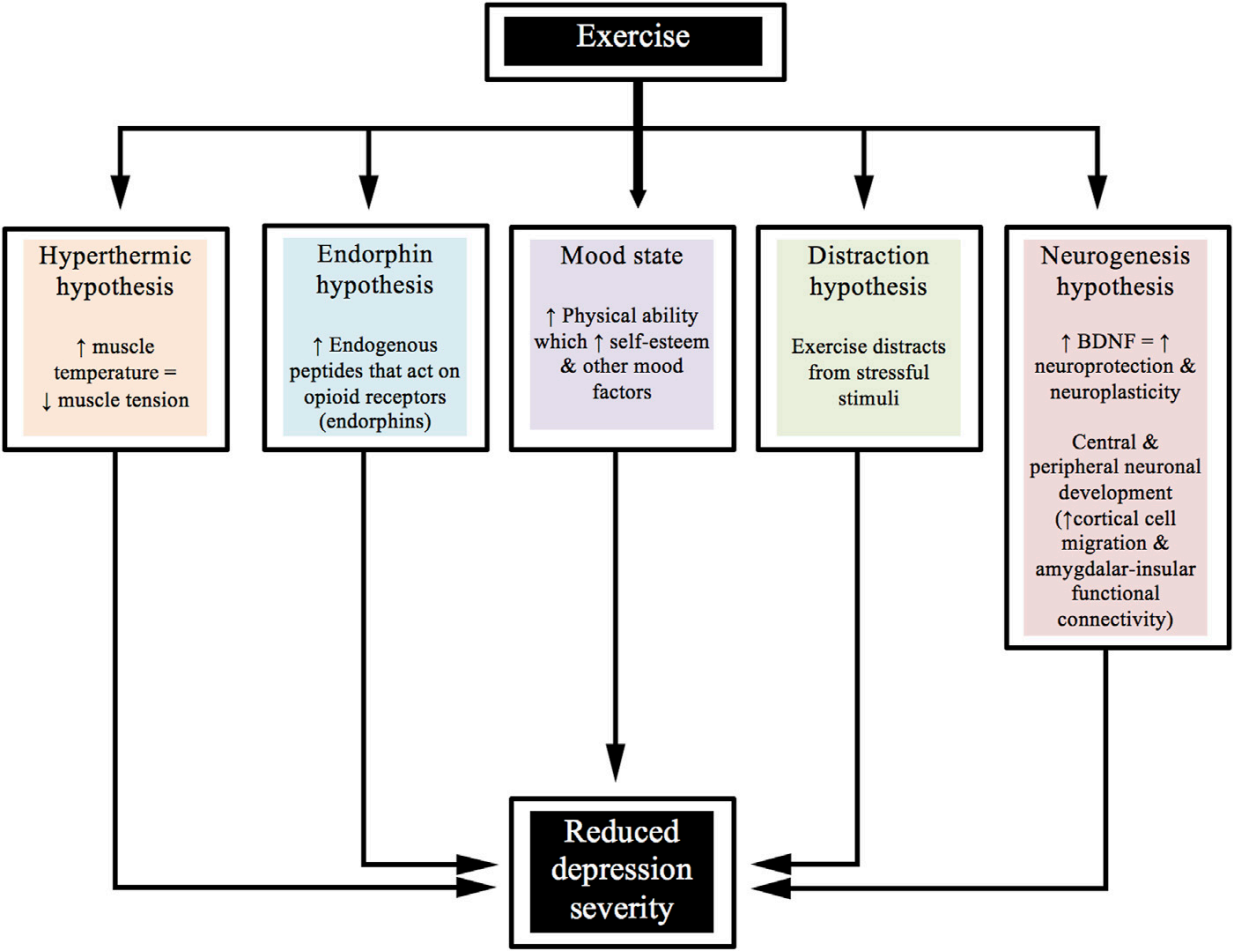
Beneficial effects of exercise on the severity of depression.

Furthermore, the combination of aerobic and resistance-based activities has been shown to be equally effective in reducing depression severity within a clinical trial by Martinsen et al. (1989). There are various ways that exercise might help to improve depression. For example, from a psychological perspective, distracting the person away from the stressful stimuli might result in a benefit. From a biological perspective, decreasing inflammation and/or increasing BDNF may be associated with such improvements. Figure 2 shows a diagrammatic representation of various hypotheses for the beneficial effects that exercise has upon the severity of depression.

### 3.2 Brain-derived neurotrophic factor (BDNF) and exercise

Seventy-six per cent of the studies reviewed found that acute forms of exercise or short-term training programs can increase BDNF concentrations in humans and 51% of the studies found significant increases in the concentration of BDNF in healthy adult subjects after acute exercise. In contrast, only 15% of the studies reported no significant increase in healthy subjects (Table 2). It is worth to mention that all the reviewed studies and/trials have reported peripheral BDNF measured in the blood stream using plasma or serum. Zoladz et al. (2008) reported that a short-term training program increased basal BDNF levels, although this study failed to include a control group. Furthermore, one study could not be classified as the reported increases in BDNF were not clearly defined as post-acute exercise or as a consequence of a training program (Coelho et al., 2012). In select clinical populations, four studies found significant increases in BDNF following acute exercise (Gold et al., 2003; Gustafsson et al., 2009; Laske et al., 2010; Strohle et al., 2010), whilst three studies failed to demonstrate an overall significant increase (Table 3) (Schulz et al., 2004; Castellano and White, 2008; Rojas Vega et al., 2008).

**TABLE 2:** Effect of exercise and other performance factors on BDNF.

Author	Age	N	Sex	Mode	Intensity	Duration	Frequency	Effect on BDNF
Castellano & White (2008)	40	11	MF	Cycling	60% VO2peak	30 min	3 x week, 8 weeks	↓acute ↑ 4 weeks
		11						↓acute
Coelho et al. (2012)	71.5	20	F	Resistance	50% 1RM week 0–2	60 min	3 x week, 10 weeks	↑ after 10 weeks
					75% 1RM week 2–10			
Correia et al. (2010)	26.4	16	M	Resistance	60 deg/s	5 sets 10 reps		No change
Ferris et al. (2007)	25.4	15	MF	Cycling	GXT	30 min	3 separate occasions	↑ +10% VT
					20% Below VT			↑Post-GXT
					10% above VT			
Goekint et al. (2008)	22.9	11	M	Cycling	55% of Wmax	60 min		↑ post exercise
					Time trial at 75% Wmax	30 min		
Goekint et al. (2010a)	20.1	15	MF	Resistance	3 sets 10 reps; 50% 1RM week 2 60%	N/A	3 x week, 10 weeks	No change
					1RM week 3–5			
					70% 1RM week 5 weeks			
					5–10 80% 1RM			
	21.5	8		No Exercise				
Goekint et al. (2010b)	21.3	9	NS	GXT: Cycling	High Intensity	2–40 min	3 x week, 8 weeks	No change
				Mixed Aerobic				
	21.2	7		No Exercise				
Goekint et al. (2011)	23.3	11	M	Cycling	55% of Wmax	60 min		↑ post exercise
					Time trial at 75% Wmax	30 min		
Gold et al. (2003)	39.2	25	MF	Cycling	60% VO_2_max	30 min		↑ post-exercise
	40.5	20						
Gomes-Osman et al. (2017)	27	12	F	Aerobic Exer	55%–64%	30 min	4 x week, 8 weeks	↑ performance on the Stroop and faster reaction times in the Continuous Performance Test detectability in the Val66Met polymorphism of the BDNF gene subgroup
								↓ in theta-burst stimulation-induced modulation of transcranial magnetic stimulation responses in Met carriers
Griffin et al. (2011)	22	32	M	GXT	Training; 60% VO_2_max	30–60 min	3 x week, 5 weeks	↑ post-exercise GXT & 5 weeks 90 min
				Cycling				
		15		No Exercise				
Gustafsson et al. (2009)	33.5	36	MF	Cycling, GXT	High Intensity	Exhaustion		↑
	34.5							↑ male controls
Håkansson et al. (2017)	65–85	19	MF	Aerobic Exer	effort corresponding to score 11–13 on the Borg rate of perceived exertion (RPE)	35 min	3 sessions	Higher working memory performance reached a higher BDNF level after physical exercise
								Single bout of physical exercise has a larger impact on BDNF than either cognitive training or mindfulness practice
Heyman et al. (2012)	23.3	11	M	Cycling	55% Wmax	60 min	1 session	↑ post exercise
					75% Wmax	30 min		
Laske et al. (2010)	NS	35	F	GXT	High Intensity	30 min		↑ post exercise
								↓ 30 min rest
		20	F					No change healthy
Levinger et al. (2008)	50.6	25	MF	Resistance	2 sets 15–20 reps at 40%–50% 1RM week 1	N/A	3 x week, 10 weeks	No change
					3 sets 8–20 reps at 50%–85% 1RM week 2–10			
	50.4	24	MF	No Exercise				
McDonnell et al. (2013)	18–60	25	MF	Cycling	Low intensity; 57% HRmax	30 min	1 session	↓ post exercise
					Moderate intensity; 77% HRmax	15 min		No change •
								↓ 5 min rest
Meyer et al. (2016)	38.6	24	F	Cycling	Low	30 min	5 sessions, 1 week apart	↑ post exercise
					Medium and High Perceived Exertion			
Murawska-Ciałowicz et al. (2015)	19–34	7	M	CrossFit	85%–95% HRmax	60 min	2 x week, 12 weeks	↑ after 12 weeks
		5	F					
Murawska-Ciałowicz et al. (2021)	27.8	35	M	Treadmill	Graded Exercise Test (GXT)	Exhaustion	First session	↑ Post GXT
Rojas Vega et al. (2006)	24.6	8	M	Cycling	High Intensity	Exhaustion		↑ Post GXT
Rojas Vega et al. (2008)	40.6	11	M	Hand biking	Moderate intensity	10 min warm-up		↑ post-warm-up
						42 km time trial		
Ross et al. (2019)	27.2	13	MF	Cycling	High intensity; 70% heart rate reserve	15 min	1 session	↑ post-exercise
								↓ 15 min rest
Saucedo Marquez et al. (2015)	27	21	M	Cycling	HIT; 90% max work for 1 min at w/1 min rest	20 min	1 session	↑ during and post-exercise
					Continuous; 70% max work			↓ 20 min rest
Schulz et al. (2004)	39.9	15	MF	Cycling	Acute; 60% VO_2_max	30 min	2 x week, 8 weeks	No change
					Training 75% Wmax			
	40	13			Acute; 60% VO_2_max		8 weeks rest	
Strohle et al. (2010)	31.9	12	MF	Treadmill	70% VO_2_max	30 min		↑ post-exercise
	30.8	12						No change
Tang et al. (2007)	19–30	16	MF	Step-exercise	Moderate	15 min		↑ post-exercise
Winter et al. (2007)	22.2	27	M	Running	Moderate intensity Exercise	40 min		
					2 × 3 min sprints			
Yarrow et al. (2010)	21.9	20	M	Resistance	Varied		5 weeks	↑ acute and post-5 weeks
Zoladz et al. (2008)	22.7	13	M	Cycling	Moderate & High Intensity		4 x week, 5 weeks	↑

M (males), F (females), GXT (graded exercise test), VO_2_max (maximal oxygen uptake), HRmax (maximal heart rate), Wmax (maximal power output) (1RM (one repetition maximum), VT (ventilatory threshold).

**TABLE 3:** Classification of participants reported in studying the effect of exercise on BDNF.

Author	Number	Healthy sedentary	Healthy active	Clinical psych	Clinical physiol	Baseline BDNF	SD	BDNF post exercise	SD
Castellano & White (2008)	11				✓(MS)	10.04(Se)	NS	NS	NS
	11	✓				20.1 (Se)			
Coelho et al. (2012)	20	✓				0.35 (P)	0.07	0.59	0.08
Correia et al. (2010)	16	✓				Legs 2.46 (P)	1.54	Legs 2.26	1.49
						Arms 1.92 (P)	1.41	Arms 1.87	1.13
Ferris et al. (2007)	15	✓				18.17 (Se)	NS	NS	NS
Goekint et al. (2008)	11		✓			Placebo 17.12 (Se)reboxetine 18.36 (Se)	3.45	NS	NS
							3.20		
Goekint et al. (2010a)	15	✓				13.6 (Se)	0.8	14.6	0.5
	8	✓				14.9 (Se)	1.4	15.4	0.7
Goekint et al. (2010b)	9	✓				NS	NS	NS	NS
	7								
Goekint et al. (2011)	11		✓			NS	NS	NS	NS
Gold et al. (2003)	25				✓(MS)	4.43 (Se)	0.5	5.95 post-30 = 4.81	NA
	20	✓				4.71 (Se)	0.49	6.76 post-30 = 4.85	NA
Griffin et al. (2011)	32	✓				Numerous measurements	−	Numerous measurements	−
	15								
Gustafsson et al. (2009)	18			✓		M = 0.487 F = 0.367 (P)	NA	30 min mean = 0.31 F = 220	NA
								60 min mean = 0.81 F = 0.76	
	18	✓				M = 0.223 F = 0.374 (P)	NA	30 min mean = 0.38 F = 0.62	NA
								60 min mean = 0.31 F = 0.60	
Heyman et al. (2012)	11		✓			12.0 (P)	NA	14.7	NA
Laske et al. (2010)	35			✓		24.4 (Se)	6.1	28.4	7.2
	20	✓				30.5 (Se)	6.9	31	8.1
Levinger et al. (2008)	25	✓				0.74 (P)	0.25	NS	NS
	24	✓				0.86 (P)	0.24		
McDonnell et al. (2013)	25	✓				162 (Se)	NA	160	NA
Meyer et al. (2016)	24			✓		15.8 (Se)	5.4	14.1 (taken within 10 min)	7.9
Murawska-Ciałowicz et al. (2015)	12		✓			Males 12.5 (Se)	4	19	5
						Females 9.5 (Se)	5	13.5	3
Murawska-Ciałowicz et al. (2021)	35		✓			Group 1,279 (Se)	143	371	NA
						Group 2,369 (Se)	241	502	
						Group 3,171 (Se)	97	236	
						Group 4,118 (Se)	34	130	
Rojas Vega et al. (2006)	8		✓			5.79 (Se)	1.9	NS	NS
Rojas Vega et al. (2008)	11	✓			✓spinal cord–injured athletes	37.2 (Se)	NS	NS	NS
Ross et al. (2019)	13			✓		31.2 (Se)	7.2	38.1	1.38
	13	✓				29.2 (Se)	6.8	36.1	1.38
Saucedo Marquez et al. (2015)	21		✓			7.96 (Se)	.448	Continuous 9.81	.581
								HIT 11.05	.588
Schulz et al. (2004)	15				✓(MS)	4.35 (Se)	3.21	5.93	5.17
	13				✓(MS)	5.08 (Se)	2.31	4.2	2.07
Strohle et al. (2010)	12		✓			NS	NS	NS	NS
	12	✓							
Tang et al. (2007)	16	✓				30.89 (Se)	8.26	34.5	8.87
Winter et al. (2007)	27	✓				NS	NS	NS	NS
Yarrow et al. (2010)	20	✓				23.3 (Se)	1.8	30.8 after acute	NA
								19.4 post-5 weeks resting	
								34.4 post-5weeks after exercise	
Zoladz et al. (2008)	13	✓				10.3 (P)	1.4	Max test 10.9	2.3
								Resting post-5 weeks 16.8	2.1
								Post-exercise 5 weeks 68.4	16.0

Se (serum), P (plasma), MS (multiple sclerosis), M (males), F (females), S (statistically significant), NS (statistically not significant), NA (not applicable), HIT (high intensity training).

#### 3.2.1 Aerobic exercise

Twenty-four studies applied an acute and/or short-term aerobic exercise ranging from a single session to 10 weeks, two to four sessions a week with varying modes, durations and intensities (Table 2, 3).

Five of those studies investigated the effects of low intensity aerobic exercise (Gold et al., 2003; Castellano and White, 2008; Griffin et al., 2011; Gomes-Osman et al., 2017; Håkansson et al., 2017). Six others looked at the effect of low-to-moderate intensity aerobic exercise (Schulz et al., 2004; Ferris et al., 2007; Goekint et al., 2008; Goekint et al., 2011; Heyman et al., 2012; McDonnell et al., 2013). Five studies investigated the effect of moderate aerobic exercise (Tang et al., 2007; Winter et al., 2007; Rojas Vega et al., 2008; Zoladz et al., 2008; Strohle et al., 2010). Two of them investigated the effects of aerobic exercise by employing both moderate and high-intensity workloads (Zoladz et al., 2008; Meyer et al., 2016), whereas six studies concentrated on the effects of high-intensity aerobic exercise only (Rojas Vega et al., 2006; Gustafsson et al., 2009; Goekint et al., 2010b; Laske et al., 2010; Saucedo Marquez et al., 2015; Ross et al., 2019; Murawska-Ciałowicz et al., 2021). Only one study investigated the combined effect of low, moderate and high intensity exercise (Meyer et al., 2016).

In addition, Saucedo Marquez et al. (2015) compared continuous (20 min at 70% Work rate max) with high-intensity interval exercise (HIIT; 90% Work rate max) on BDNF release and recovery in physically active young men. One study that failed to report the intensity of workload over a training program (Goekint et al., 2010a). Ferris et al. (2007) found increases in BDNF following more strenuous exercise (75% VO_2_max), compared with exercise at 20% below ventilatory threshold (56% VO_2_max) which yielded no significant change, suggesting that BDNF response may be exercise intensity-dependent.

This finding was also supported in later studies reported by Saucedo Marquez et al. (2015) and Ross et al. (2019). Saucedo Marquez et al. (2015) found that higher intensity exercise was slightly more effective vs continuous exercise in elevating serum BDNF concentrations whereas Ross et al. (2019) compared high-intensity to low-intensity, finding that only high-intensity exercise produced significant increases in BDNF. Although, studies have found increased BDNF with high intensity exercise (e.g., Rojas Vega et al., 2006; Ferris et al., 2007; Goekint et al., 2008; Gustafsson et al., 2009; Laske et al., 2010; Griffin et al., 2011; Heyman et al., 2012) other studies within this review have shown that moderate intensity exercise can also significantly increase BDNF concentrations following acute exercise (e.g., Schulz et al., 2004; Tang et al., 2007; Goekint et al., 2008; Rojas Vega et al., 2008; Gustafsson et al., 2009). Ross et al. (2019) confirmed the linear relationship between cortisol levels and BDNF following exercise.

Zoladz et al. (2008) reported a training effect upon peripheral BDNF, and that the majority of the prescribed exercise intensity was low-moderate; however, the program incorporated both low-moderate and very high-intensity training (∼90 $\dot{V}O$ _2max_).

The only aerobic exercise-based study that found that exercise significantly decreased BDNF level after an acute exercise bout was Castellano & White (2008). The decrease was noticed in both healthy controls and patients with multiple sclerosis between baseline measurements and 2 h post-exercise, and between baseline measurements and 3 h post-exercise at weeks 0, 4 and 8. The decreases in BDNF observed after acute exercise are likely explained by the collection of blood 30 min after exercise cessation, whilst all other studies that collected blood post-exercise did so immediately, though in some studies there were subsequent collections during post-exercise periods. Heyman et al. (2012) revealed and Ross et al. (2019) confirmed that while BDNF levels are substantially elevated immediately after an acute bout of exercise, they drop to at or below baseline levels within 15 min of exercise cessation. This clearly indicates the necessity of timing of sample collection in the validity of studies examining the relationship between BDNF and exercise. Further, there was a linear relationship between the mean heart rate over the 15-min bout of exertion and subsequent BDNF levels immediately post-exercise (Ross et al., 2019).

The correlation to heart rate rather than anaerobic exertion is consistent with the findings of Murawska-Ciałowicz et al. (2021). These latter showed BDNF levels increased immediately following intense aerobic exercise performed as the Graded Exercise Test (GXT), but not after the Wingate Anaerobic Test (WAnT). The GXT involves running on a treadmill at 6 km/h, but increases in 2 km/h increments every 3 min until the subject reaches exhaustion and is typically used to measure maximal oxygen uptake (VO_2_max). The WAnT, on the other hand, involves 30 s of maximal effort on a cycle ergometer. Not only did this study confirm the need for the intensity to be great enough to maximize heart rate and oxygen utilization, but its primary aim was to determine if 9 weeks of high-intensity training would change baseline (resting) levels of BDNF (Murawska-Ciałowicz et al., 2021). The same author has previously demonstrated that 12 weeks of crossfit training have significantly elevated the resting serum BDNF levels in active men and women together with several other fitness and body composition biomarkers (Murawska-Ciałowicz et al., 2015). The participants performed 1 hour crossfit split into two parts (power training and aerobic training), twice per week where the intensity was kept between 85% and 95% HRmax. Although 15 males and 15 females started the investigation, only seven and five, respectively, completed the entire investigation. This dropout rate could have affected further BDNF data collected after the GXT and the Wingate tests post the 3 months crossfit training which were inconsistent between both genders (Murawska-Ciałowicz et al., 2015).

Four different methods of high-intensity training were examined and none changed resting BDNF after 9 weeks of training (Schulz et al., 2004). The study did find that aerobic exercise training increased BDNF during week 4 at rest compared to rest at baseline in patients with multiple sclerosis, yet tended to decrease back towards baseline values after 8 weeks of cycle ergometer training (at 60% $\dot{V}O$ _2_ peak). This was the only study that measured BDNF concentration during the mid-point of a training regime and which reported BDNF fluctuations during a training program. Later studies (Gilder et al., 2014; Saucedo Marquez et al., 2015) confirmed a rapid return to baseline levels for BDNF immediately on cessation of exercise.

An interesting finding was that serum BDNF concentration in the study of Rojas Vega et al. (2006) was comparatively lower than other studies employing healthy adults, though it should be noted that this study recruited recreational athletes. Studies using well-trained subjects have reported lower resting serum BDNF levels (e.g., Chan et al., 2008), although some studies with well-trained, young cyclists have not observed similar lower concentrations (Goekint et al., 2008). Lower BDNF at rest, observed in well-trained individuals, has been proposed to be the result of higher, more effective clearance rates of circulating BDNF (Knaepen et al., 2010; Gilder et al., 2014). These findings correlate with an inverse relationship discovered between the resting peripheral BDNF level and long-term physical exercise as well as cardiorespiratory fitness; whereby there was a positive correlation between BDNF and age with the peripheral BDNF levels increasing as we get older (De la Rosa et al., 2019). In a recent large European cohort, higher plasma BDNF levels at baseline were found to be associated with being older, female sex, and higher prevalence of smoking, whereas the plasma BDNF was found not to be associated with total physical activity level or the amount of moderate to vigorous activity (Galle et al., 2021).

Two studies measured the effect of a warmup upon serum BDNF concentrations (Rojas Vega et al., 2006, 2008), with only Rojas Vega et al. (2008) finding significant increases post-warmup. The 2008 study involved spinal cord injured athletes; whereas the author’s earlier study used healthy subjects, although the 2006 study did not report the exact intensity of the warmup protocol (‘moderate exercise’) and did not include a methods section within the report.

A year-long, randomized study (walking vs. stretching) in men and women with a mean age of 66.4 years showed no significant difference in the BDNF concentration (Voss et al., 2013). Non-etheless, changes in serum BDNF were positively associated with markers of neural plasticity (Voss et al., 2013; Leal et al., 2017). Similarly, a six-month-long training program in older subjects (age range 63–80 years) which compared ‘dance’ (an environmentally enriched program) to ‘sport’ (as an exercise intervention) showed an increase in plasma BDNF levels only for the dance group, together with larger volume increases in, e.g., corpus callosum and the sensorimotor cortex. However, the study failed to report when BDNF measures were made, nor the absolute concentrations of either plasma or serum BDNF (Rehfeld et al., 2018).

#### 3.2.2 Resistance exercise

Only five studies were found that investigated the effects of acute and/or short-term resistance exercise upon BDNF concentrations levels (Levinger et al., 2008; Goekint et al., 2010a; Correia et al., 2010; Yarrow et al., 2010; Coelho et al., 2012), three of which followed an exercise training program ranging from five to ten weeks, three exercise sessions per week incorporating a variety of sets, resistance and repetitions (Levinger et al., 2008; Goekint et al., 2010a; Yarrow et al., 2010). Corriea et al. (2010) was the only team that used isokinetic dynamometry throughout, although Coelho et al. (2012) incorporated isokinetic dynamometry to assess muscle strength. Yarrow et al. (2010) was the only study that incorporated two different resistance-based programs for comparison. The authors followed a progressive resistance program on the bench press and squat exercise, with increasing workload over time; either four sets of six reps at 52.5%–75% 1RM (one repetition maximum) of eccentric and concentric exercise or three sets of six reps at 40%–50% 1RM concentrically and 100%–120% 1RM eccentrically. This study found that both intensities of exercise increased BDNF to a similar magnitude; however, BDNF levels measured after the resistance training protocol were actually lower than baseline before any exercise.

Similarly, Coelho et al. (2012) found that a resistance training program with intensities of 50%–75% of one repetition maximum (1RM) can elicit a positive increase in BDNF. However, this study did not clearly state whether BDNF was analysed after training for 10 weeks at baseline, and therefore it is difficult to determine if the observed increase was due to short-term training effect or merely a consequence of acute exercise. Further, this paper did not clearly state if BDNF was measured immediately after an acute bout of exercise. Three studies did not find significant changes in BDNF following acute and/or short-term resistance-based exercise (Levinger et al., 2008; Correia et al., 2010; Yarrow et al., 2010). The study by Goekint et al. (2010a) involved a progressive resistance program, incorporating three sets of ten repetitions ranging from 50% 1RM to 80% 1RM. Six different exercises were performed using upper body and lower body muscles. The authors suggest that the failure to increase BDNF in their study was potentially due to the training regime not being intense enough to induce biochemical changes. However, it is evident that subjects were exposed to high-intensity workloads of 80% 1RM for a period of 5 weeks and thus their conclusion is somewhat questionable. It is also worth noting that the study had a low number of participants (*n* = 11). In addition, the study by Corriea et al. (2010) required subjects to exert force against a lever as the study used an isokinetic dynamometer; the force exerted depended upon the subjects’ motivation, and this may have influenced the optimal response of BDNF if subjects failed to exert maximal force during the whole course of exercise.

Another factor regarding BDNF and resistance exercise may be associated with lactate. Immediately following high-intensity exercise, peripheral lactate levels substantially increased, which were associated with increased peripheral BDNF levels (Levinger et al., 2008). Lactate produced during exercise has been shown to be able to cross the blood-brain barrier *via* monocarboxylate transporters, which are proton-linked membrane carriers involved in the transport of monocarboxylates, including lactate, pyruvate, and ketone bodies (Pierre and Pellerin, 2005). The transportation of lactate across the brain-blood barrier may be the necessary link correlating how physical exercise is directly involved in the BDNF-dependent neurobiological pathways (silent information regulator 1 (SIRT1)-dependent induction of the PGC1α/FNDC5pathway) (El Hayek et al., 2019). In addition, studies have demonstrated that lactate infusion at rest was able to increase peripheral and central BDNF levels as well (Müller et al., 2020).

### 3.3 Effects of exercise on brain neuroplasticity

Neuroplasticity is the brain’s inherent capacity to change and adapt due to experience. It is an umbrella term that recognises the brain’s ability to change, reorganize, or grow neural networks. This can involve functional changes due to brain damage (hence the brain moves functions from a damaged area of the brain to other undamaged areas), or structural changes as a result of learning (hence the brain changes its physical structure) (Sebastianelli, et al., 2017). Recently, research has demonstrated that the brain continues to create new neural pathways and alter existing ones in order to adapt to new experiences, learn new information, and create new memories (Sebastianelli et al., 2017).

Disruptions in neuroplasticity pathways may contribute to the pathophysiology of depression (Pittenger and Duman, 2008). Therefore, targeting neuroplasticity could become a potential novel treatment approach (Kays, et al., 2012). Exercise could be one such approach as it appears to effectuate central neuroplastic adaptation *via* optimisation of BDNF levels (Phillips, 2017). An optimised BDNF level could be achieved *via* a combined therapy that uses antidepressants and physical activity in patients with major depressive disorder. It could optimize BDNF in key brain regions to promote neuronal health and functional recovery in their key circuits (Phillips, 2017). However, the challenge that is still to be solved is to identify how exercise and physical activity could boost the neuroplasticity mechanisms of the brain structure and function. According to the same author, this challenge will necessarily require a better understanding of how the optimum mode, intensity, and duration of exercise and physical activity might alter major depressive disorder related symptoms and pathology. Exercise enhances BDNF release, promoting dendritic spine integrity and activating other pathways that contribute to plasticity (Phillips, 2017). Exercise appears to increase the volume of both the left and right portions of the hippocampus and several cortical regions in healthy participants (Li et al., 2016; Firth et al., 2018; Zheng et al., 2019). Naveen et al. (2016) compared BDNF and cortisol levels in three groups of patients with depression. They found that reduced cortisol values were inversely correlated with the increased BDNF levels solely in the group that was treated with yoga only, unlike groups that were treated with yoga + antidepressant or antidepressant only. Nevertheless, we should mention that the study involved only small groups (54 subjects in total; with the antidepressant-treated n = 16, yoga + medication treated n = 19, and yoga only n = 19). In addition, patients were using different types of antidepressants (escitalopram, fluoxetine, duloxetine, sertraline, amitriptyline, mirtazapine), which might have affected BDNF and cortisol results.

Heyman et al. (2012) provided experimental-based evidence that acute intense exercise increases the peripheral levels of anandamide (AEA) and 2-arachidonoylglycerol, (2-AG) and that BDNF might be a mechanism by which AEA influences the neuroplastic and antidepressant effects of exercise. Although their study involved healthy subjects who undertook intense 90 min cycling exercise, it is worth mentioning that AEA levels increased during exercise and post 15 min recovery, whereas 2-AG levels were unchanged. However, BDNF levels increased during exercise and then decreased post 15 min of recovery. Remarkably, AEA and BDNF were positively correlated at the end of exercise and after the 15 min recovery. In addition, AEA production during exercise have been stimulated by cortisol since a positive correlation was found between both compounds and because corticosteroids are known to stimulate endocannabinoid biosynthesis. The authors suggested that the increase of the AEA during exercise might be one of the factors that trigger exercise-induced increase in peripheral BDNF and that the high levels of the AEA during recovery might also delay the return of BDNF to basal levels.

### 3.4 Cognitive response to exercise

Impairment in cognitive functioning has also been associated with major depression (Baune et al., 2010). Exercise has been effectively shown to enhance learning and memory formation as well as alleviate the symptoms of depression mediated by inducing BDNF expression and signalling in the hippocampus and other brain regions (El Hayek et al., 2019). BDNF levels after one bout of exercise were shown to be dependent on duration time, intensity, and type of test/exercise and correlated to lactate and minute ventilation (Murawska-Ciałowicz et al., 2021). BDNF levels have been shown to increase as much as two to three-fold immediately following acute exercise in humans when compared to at rest conditions, whereby these elevated levels of BDNF also positively correlated with improvements in cognitive functions (De la Rosa et al., 2019). Some studies investigated the cognitive response to exercise (Ferris et al., 2007; Goekint et al., 2010a; Goekint et al., 2008; Goekint et al., 2010b; Griffin et al., 2011; Winter et al., 2007). Ferris et al. (2007) found that moderate and higher intensity exercises can positively influence cognitive domains related to attention, processing speed and executive function as measured by the Stroop colour and word test, although the study failed to find a correlation between these cognitive domains and an increase in BDNF. Similarly, and more recently, Slusher et al. (2018) showed that an acute and short session of supramaximal high-intensity interval training significantly increased the prefrontal cortex-dependent executive function in a randomized, counterbalanced study. The healthy male participants performed the Wisconsin Card Sorting Task immediately following a 5-min seated rest (which served as control) and after an exercise protocol undertaken 2 weeks apart. The supramaximal high intensity interval training consisted of ten maximal bouts of all-out pedalling on a cycle ergometer for 20 s (separated by 10 s of active recovery) against 5.5% of the body weight. Slusher et al. added that the improvements observed in prefrontal cortex-dependent executive function were independent of BDNF concentrations in plasma and serum. However, Griffin et al. (2011) failed to find significant alterations in Stroop word-colour task after acute exercise (graded exercise test to exhaustion-GXT), 3 weeks, or 5 weeks of moderate-intensity aerobic training, although improvements in a memory test (face-name matching) and, importantly, BDNF concentration were reported after acute exercise and at 5 weeks.

In another study, Goekint et al. (2008) investigated short-term memory (Digit Span memory test) & mid-term memory (picture recall and recognition) in well-trained male cyclists. They found that mid-term memory was impaired after acute aerobic exercise, although short-term memory was unchanged. In contrast, Goekint et al. (2010a) suggested that a short-term resistance exercise program can significantly improve short-term memory (Digit Span) in healthy untrained subjects, whereas mid-term memory (picture recall) was unaffected by exercise. Since these improvements in memory were found in both the experimental and control groups, the authors suggested that the improvements may be due to a learning effect. In addition, there was also a positive influence on BDNF concentrations, yet this study did not perform any correlations between changes in BDNF and the observed enhancements in short-term memory to investigate whether a transient increase in the BDNF was linked with enhancements in memory components. Another study by Goekint et al. (2010b) found no influence of short-term aerobic exercise upon either short-term or mid-term memory tests in untrained individuals, although the authors implemented the identical tests used in the study by Goekint et al. (2010a). An earlier study by Winter et al. (2007) did, however, find a positive correlation between increased levels of BDNF and enhanced short-term learning following high-intensity anaerobic sprinting. The authors found that high-intensity rather than low-intensity aerobic exercise significantly raised BDNF levels post-exercise.

### 3.5 Antidepressant treatment and acute and short-term exercise

The longest antidepressant treatment reported in the literature was 1 year (Piccinni et al., 2008a) whilst, for exercise, it was 10 weeks (Levinger et al., 2008; Goekint et al., 2010a; Coelho et al., 2012). The course of antidepressant action upon peripheral BDNF was not significantly altered at one, three, six and 12 months of administration in the study by (Piccinni et al., 2008b; Goekint et al., 2010a) also found no differences with a comparatively longer strength-training program; however, the author concluded that the intensity and workload used might not have been sufficient to bring about a notable change in peripheral BDNF. Most of the studies investigated the BDNF response to exercise after a single session, of which only seven studies failed to find a significant increase in the BDNF concentration (Schulz et al., 2004; Castellano and White, 2008; Levinger et al., 2008; Correia et al., 2010; McDonnell et al., 2013; Meyer et al., 2016). The effects of acute physical activity and a training program were reported in a few studies that mostly focused on training effects. As discussed within the aerobic exercise section, only one study reported a training effect upon basal BDNF levels (Zoladz et al., 2008), although a study by Coelho et al. (2012) failed to clearly report the timescale of BDNF measurement (acute or training effects). The largest increases in peripheral BDNF reported after an exercise intervention of 5 weeks was that of Zoladz et al. (2008), from 10.3 ± 1.4 pg/mL to 68.4 ± 16.0 pg/mL (plasma), whereas for antidepressant treatment it was 29.4 ± 12.6 ng/mL to 52.3 ± 12.7 ng/mL (serum) after 6 months treatment (Matrisciano et al., 2008).

Meyer et al. (2016) found that self-reported improvements in depression symptoms were significantly improved after an acute bout of exercise lasting 30 min and that the degree of change thereof was not intensity dependent. They found no correlation between BDNF and symptom improvement; however, they described the BDNF sampling as occurring “within 10 minutes” of the cessation of exercise. As described earlier, there is such a dramatic drop in BDNF levels within the first 15 minutes of stopping that any heterogeneity in collection times spanning 10 minutes would render a correlation to symptoms worthless (Ross et al., 2019).

Piccinni et al. (2008b) reported decreases in serum BDNF after antidepressant treatment from 19.3 ± 8.8 ng/mL to 18.8 ± 4.1 ng/mL, although the study had a large subject dropout. Inversely, many other authors found significant increase. Interestingly, Aydemir, et al. (2005) and Aydemir, et al. (2006) had younger patients (with likelihood of better plasticity of the brain) that demonstrated the highest post-treatment serum BDNF increase when compared to other studies with older subjects. The same authors showed significant reduction in the severity of depression after antidepressant treatment. However, Aydemir, et al. (2005) increased the dose of antidepressants for five patients and noted the associated improvement before the final assessment. Therefore, the dosage administration throughout the trials was not standardised, which could have affected the results; Yarrow et al. (2010) found a decrease in BDNF at baseline after 5-week of resistance training from 23.3 ± 1.8 ng/mL to 19.4 ng/mL; however, this study found significant post-exercise increases in BDNF; Schulz et al. (2004) also found reduced BDNF levels after a period of 8 weeks rest in multiple sclerosis control subjects from 5.1 ± 2.3 ng/mL to 4.2 ± 2.1 ng/mL, however, the exercise group of patients with multiple sclerosis showed slight increases with an exercise regime from 4.3 ± 3.2 ng/mL to 5.9 ± 5.2 ng/mL. In addition; Castellano & White, (2008) found acute exercise reduced BDNF, although there were blood measurement issues as discussed earlier (aerobic exercise section).

The majority of exercise-based studies report that BDNF levels following acute exercise return to baseline values 15–60 min post-exercise cessation, although two studies found significantly lower BDNF concentrations during rest of 2–3 h when compared to basal BDNF values (Castellano and White, 2008; Yarrow et al., 2010). In fact, a recent 9-week exercise study found that higher pre-exercise BDNF levels were indeed found to be a significant factor involved with the exercise-induced improvements related to frontal lobe function, but was independent of the exercise-induced alterations in resting peripheral BDNF levels, implying that exercise is able to induce improvements in frontal lobe function but not in inducing long-term changes in the peripheral BDNF (da Silveira-Rodrigues et al., 2021). Could this rapid return to baseline values indicate re-uptake by the brain to exert BDNF’s neuroplastic effects?

### 3.6 Antidepressant treatment *versus* exercise; issues relating to side effects

There are a number of side effects associated with antidepressant treatment, which depend upon various factors including those related to drugs (type, dosage) and the length of antidepressant administration as well as individual genetic variation and the patient’s medical factors, such as the presence of any chronic disease(s) and the state of renal and hepatic functions (Cascade et al., 2009). Many antidepressants produce some antisialagogue effects, causing ‘dry mouth’ by decreasing saliva production (Wimbiscus et al., 2010). Weight gain is also observed during treatment with some tricyclic antidepressants (TCAs), and more modern antidepressants such as Mirtazapine. SSRIs may initially induce weight loss during treatment although during long-term treatment weight can be gained (Zimmermann et al., 2003). In a review by Cascade et al. (2009) evaluated the effects of SSRIs in 700 patients, patients reported weight gain as one of the most common side effects.

Some antidepressants have been reported to cause impairments in glucose homeostasis (Fisfalen and Hsiung, 2003; Carvalho et al., 2004)^.^ Another review into the association of antidepressant usage and risk for diabetes found that long-term, moderate doses of SSRIs and TCAs increased the risk of diabetes in patients (Andersohn et al., 2009). Several studies have suggested that the use of SSRIs is associated with an increased risk of bleeding, particularly upper gastrointestinal bleeding (Dalton et al., 2003; Meijer et al., 2004; Vidal et al., 2008; Van Haelst et al., 2010). Furthermore, SSRIs have been associated with an increased risk of haemorrhagic and fatal stroke (Smoller et al., 2009), although Douglas et al. (2011) found no evidence that SSRIs are associated with haemorrhagic stroke. In addition, clinical studies have shown that SSRIs use can reduce bone mineral density (Haney et al., 2007) and increase the risk of bone fracture (Cizza et al., 2010; Ensrud et al., 2003). A meta-analysis by Cizza et al. (2010) investigating the role of SSRIs in bone turnover, proposed that depression induces bone loss and osteoporotic fractures, primarily *via* specific immune and endocrine mechanisms, with poor lifestyle habits such as physical inactivity acting as potential contributory factors. Further, it indicated that major depressive disorder was associated with lower bone mineral density in the spine, femur, and femoral neck (Cizza et al., 2010). The authors concluded that deficits in bone mineral density in subjects with depression were of clinical significance and likely to increase fracture risk over the lifetime of these subjects, suggesting this could be due to the inactivity associated with depression. Earlier studies confirmed sexual dysfunction is a common side effect of antidepressant treatment (Gregorian et al., 2002). Other commonly reported adverse effects of antidepressants include dizziness, nausea, insomnia, drowsiness, fatigue, and tremors. Thus, it appears that either depression or certain antidepressant treatments can lead to the loss of bone mineral density. Physicians and other medical professionals may, therefore, consider alternative types of antidepressants when their patients are at an increased risk of osteoporosis.

It is suggested that the outcome of treatments for Major Depressive Disorder using currently available antidepressants is rather disappointing as only 3%–40% of “real life” patients experience remission with the first antidepressant selected (Warden et al., 2007). There are also the cost issues surrounding antidepressant treatment. It also should be noted that antidepressants, in general, should be continued for at least 6 months after the remission of symptoms for the first episode of depression and if the patient has a history of previous episodes of depression, the antidepressant treatment is recommended to continue for about 2 years.

From the other side, exercise has fewer side effects that are generally minor. Obviously, injuries could happen but rarely for those who undertake a supervised training regime for health. Access to the gym could be costly in some places but patients are generally subsidised by their local health authorities. Some wealthy patients could buy their own equipment/gym enabling them to work out in the comfort of their own homes.

## 4 Physiological mechanism

Shimizu et al. (2004) have shown that a single nucleotide polymorphism resulting in a substitution of an adenine (A) base by guanine (G) in the BDNF gene (BDNF) has been identified in approximately 20% of the human population. This polymorphism is designated as “Val66Met” due to the substitution of the Valine to Methionine in the 66th amino-acid position of the synthesized BDNF with respect to the A or G genotype. Nonetheless, it has been suggested that the BDNF Val66Met may play a role in the aetiology of several neurological diseases (Notaras and van den Buuse, 2018; Shen et al., 2018) and psychiatric disorders (Kishi et al., 2018).

To the best of our knowledge and to date, the only paper that demonstrated that the two alleles of human BDNF are expressed simultaneously in both non-polymorphic and polymorphic humans, carrying Val66-and Met66-coding alleles was published by De Assis et al. (2021). Their investigation which included muscle biopsies and genotyping pre and post GXT in 13 healthy males showed that the expression of BDNF immediately after maximal exercise decreases 1.8-fold, regardless of the genotype (Table 4). It also showed that BDNF expression from the Met66-coding alleles, in heterozygotes, was 1.3-fold lower than that from the Val66-coding alleles. However, the same authors added that the plasma BDNF has increased by the metabolic stress immediately induced by the GXT but without any correlation with the BDNF expression. De Assis et al. (2021) suggested that muscle BDNF expression is very stable and that the changes observed following exercise are not dependent on the participants’ age or genotype. It is likely that the preliminary decline in BDNF expression noticed during acute metabolic stress, is not only another proof of the stability feature for BDNF expression, as well as its immediate sensitivity to metabolic changes but would also be followed by a compensatory rise during recovery. The authors alluded that there might be a tendency to maintain the plasma circulating BDNF concentrations within a certain biological range during metabolic stress, such as exercise (Table 4).

**TABLE 4:** Suggested physiological mechanisms underpinning BDNF modulation.

	Muscle	Blood	Brain
Light Exercise	Increased peripheral BDNF and lactate	More significant increased peripheral BDNF and a parallel increased of lactate	Pierre and Pellerin (2005)
			Lactate able to cross the BBB *via* MCT’s
			Müller et al. (2020)
			- Lactate infusion increases central BDNF and plays an important role in exercise induced neuroplasticity
			- Lactate binding to HCAR1 at the BBB boosts the development of new blood vessels
			- Lactate could either bind directly to the HCAR1 or transmembrane *via* MCT’s in the neurons to achieve a neurotrophic and metabolic effects
			- Lactate inhibits the (AC) and decreases cAMP in the neurons, which would reduce BDNF expression and the subsequently the regulatory function in the control of blood flow, and synaptic functions
			- Lactate can stimulate the (PGC1α/FNDC5/BDNF) pathway through SIRT1 activation
			- Lactate could increase intracellular NADH, which could increase Ca^2+^ levels, hence BDNF gene expression
			- BDNF production boosts neuroplasticity through neurogenesis, synaptogenesis, growth of dendritic spines, long-term potentiation, etc
			Jimenez-Maldonado et al. (2018)
			- HIIT increases the NMDA receptor activity to raise intracellular Ca^2+^ concentration in neurons
			- Ca^2+^ activates the CaMKII activity and the MAPK/ERK/MSK signalling which would prompt the Creb-BDNF transcription and neuronal plasticity
			- Ca2+ increases the ROS production intra-neurons. Once synthetized, ROS can activate Creb-BDNF transcription
Intense Exercise	De Assis et al. (2021)	De Assis et al. (2021)	
	- BDNF expression decreases immediately after GXT about 1.8-fold, regardless of the genotype	- Plasma BDNF increases immediately after GXT but without any correlation with the BDNF expression	
	- BDNF expression from the Met66-coding alleles is 1.3-fold lower than that from the Val66-coding alleles	- Tendency to maintain the plasma circulating BDNF concentrations within a certain biological range during metabolic stress	
		Brunelli et al. (2012)	
		- BDNF circulating within the peripheral blood mononuclear cells could be regulated by physiological stress caused by exercise	
		- Blood solutes induced by hemoconcentration change because of the alteration in blood volume rather than a real exercise-induced change in BDNF levels	
		- Increased intracellular BDNF immediately after a GXT to a during the recovery period would	
		- GXT increases the of p75^NTR^ expressed within the peripheral blood mononuclear cells, indicating that BDNF isoforms are produced and secreted by the immune cells to modulate the apoptotic pathway when the stressors’ intensity reached a certain level	
		Heyman et al. (2012)	
		- AEA and 2-AG increase and BDNF might be a mechanism by which AEA influences the neuroplastic and antidepressant effects of exercise	
		- AEA production stimulated by cortisol	
		- Increased AEA during exercise might be one of the factors that triggers exercise-induced increase in peripheral BDNF.	
		Jimenez-Maldonado et al. (2018)	
		- HIIT elevates systemic blood lactate concentration, and consequently increases the NMDA receptor activity to raise intracellular Ca^2+^ concentration in neurons	
Post Exercise (recovery)	De Assis et al. (2021)	Brunelli et al. (2012)	
	BDNF expression compensatory rise	- BDNF fast decrease at 30 min and 60 min post GXT explained by BDNF release from peripheral blood mononuclear cells, with also a possible use by the cell itself	
	Castellano and White (2008); Yarrow et al. (2010)	- BDNF accumulation post moderate intensity exercise indicates an adaptive response to hormone stress condition	
	Lower BDNF concentrations post 2–3 h when compared to basal values	Heyman et al. (2012)	
		- AEA increases post 15 min recovery, whereas 2-AG unchanged	
		- BDNF decreases post 15 min recovery	
		- The high levels of AEA might delay the return of BDNF to basal levels	

There is accumulating evidence showing that although the brain is the main source of systemic BDNF during exercise, other peripheral sources might modulate their BDNF expression in response to exercise. Apart from Rasmussen et al. (2009), Brunelli et al. (2012) for instance, showed that the level of BDNF circulating within the peripheral blood mononuclear cells could be regulated by physiological stress caused by exercise (Table 4). However, it is worth noting that their suggestion was based on previous studies and not on experimental findings. Indeed, previous studies demonstrated that under stress conditions immune cells were capable to produce and release BDNF (Schulte-Herbrüggen et al., 2005).

Brunelli et al. (2012) further suggested that moderate-to-intense acute exercise could decrease the volume of circulating plasma. The changes in blood solutes they have measured after their exercise sessions could be related to a hemoconcentration change because of the alteration in blood volume rather than a real exercise-induced change in BDNF concentration. They also recommended that studies investigating the effect of exercise on the circulating BDNF should be standardised. The same authors went further to speculate that, the transition from the increased intracellular BDNF immediately after a GXT to a fast decrease at 30 min and 60 min during the recovery period would involve BDNF release from peripheral blood mononuclear cells, with also a possible use by the cell itself. Contrarily, the BDNF accumulation observed during the recovery phase that follows moderate intensity exercise could indicate an adaptive response to hormone stress condition Brunelli et al. (2012), (Table 4).

TrkB is the high-affinity receptor of the BDNF, however, the latter has also a low-affinity receptor (p75^NTR^) that could be involved in the apoptotic pathway by binding with pro-BDNF. TrkB conversely, could be involved in the pro-survival effects Brunelli et al. (2012). The GXT exercise protocol (but not moderate or light exercise) increased the amount of p75^NTR^ expressed within the peripheral blood mononuclear cells which could indicate that BDNF isoforms are produced and secreted by the immune cells to modulate the apoptotic pathway in autocrine and/or paracrine manner when the stressors’ intensity reached a certain level (Brunelli et al., 2012).

The above sections have provided evidence of the interplay between an increased peripheral BDNF level and a parallel increase of lactate, in particular following high-intensity exercise. Lactate infusion has indeed been shown to increase peripheral and central BDNF and plays an important role in exercise-induced neuroplasticity (Müller et al., 2020). The authors suggested a few mechanisms underpinning this phenomenon.

It has been shown in section 2.2, that lactate can cross the blood-brain barrier (BBB) *via* several monocarboxylate transporters (MCT’s). Lactate binding to the hydroxycarboxylic acid receptor (HCAR1) at the BBB would induce/enhance the development of new blood vessels. Once in the neuron, lactate could either bind directly to the HCAR1 or be transported through the membranes *via* MCT’s to achieve a number of neurotrophic and metabolic effects, such as conversion to pyruvate (Müller et al., 2020), (Table 4).

One of the mechanisms that the same authors put forward is that lactate binding to HCAR1 at the neurons could inhibit adenylate cyclase (AC) and could consequently decrease cAMP, which would reduce BDNF expression and the subsequently the regulatory function in the control of blood flow, and synaptic functions. The second suggested mechanism is that lactate can stimulate the peroxisome proliferator-activated receptor γ co-activator α/fibronectin type III domain-containing 5/BDNF (PGC1α/FNDC5/BDNF) pathway through silent information regulator 1(SIRT1) activation. The third suggested mechanism is that lactate could increase intracellular NADH to assist in pyruvate conversion, which could increase calcium levels, hence BDNF gene expression. The produced BDNF can then boost neuroplasticity through a number of neurobiological mechanisms (such as neurogenesis, synaptogenesis, growth of dendritic spines, and long-term potentiation).

Similar to the above, Jimenez-Maldonado et al. (2018) suggested three theories underpinning the effect of high-intensity interval training (HIIT) on BDNF, amongst them the following could be the most realistic based on the above sections and also on the latest reported findings (Table 4): HIIT elevates systemic blood lactate concentration and consequently increases the NMDA receptor activity to raise intracellular Ca^2+^ concentration in neurons. The ion activates the calcium/calmodulin-dependent protein kinase II (CaMKII) activity and mitogen-activated protein kinase/extracellular signal-regulated kinase/mitogen- and stress-activated kinases (MAPK/ERK/MSK) signalling which would prompt the cAMP-response element binding protein/BDNF (Creb-BDNF) transcription and neuronal plasticity. Moreover, the intracellular Ca^2+^ could increase the reactive oxygen species (ROS) production intra-neurons. Once synthesised, ROS can activate Creb-BDNF transcription (Jimenez-Maldonado et al., 2018).

## 5 Limitations

There is a paucity of studies that investigated the effect of resistance exercise on BDNF compared to the ones investigated the effect of aerobic exercise. A major limitation of this review is the limited number of studies that analysed depression severity and BDNF in response to exercise in patients with clinical depression. Until this gap is filled, we feel that the studies we assessed do not do enough towards bridging the hypothetical link between the short-lived post-exercise rise in BDNF and improvements in depressive symptoms. Studies should attempt to compare depression severity from transient increases and long-term increases by investigating a variety of short-term exercise programs, including a consideration of the factors that affect the levels of BDNF in humans and in patients. Moreover, it would be interesting and important to compare the effects of exercise and antidepressants on self-reported severity of depression such as, “Does exercise reduce depression *via* a transient increase in BDNF to a greater degree than antidepressants?”

Another limitation of this study is the fact that we only checked three databases. Although articles could be mentioned in several search engines, interrogating further research databases to capture more relevant papers (including other languages) could also be appropriate. This could better clarify the underpinning concepts, investigate the methodological quality of the studies, set some inclusion/exclusion criteria, further appraise the quality of the evidence, and answer potential questions *via* wider institutional consensus.

It appears that the methodology of collecting and measuring BDNF is exceedingly significant to the conclusions that could be drawn as serum BDNF levels could be 25 to 120 times higher than plasma, making a side-by-side comparison very challenging.

## 6 Conclusion

The aim of this extensive review was to explore the relevant neurobiology and the association between peripheral levels of BDNF and acute and short to long-term exercise regimes, as well as its relationship to depression and antidepressant treatment. This review shows that acute exercise, particularly high-intensity exercise, elevates BDNF levels in healthy humans and clinical populations, as evidenced by aerobic and resistance-based studies. More importantly, there is an increase in BDNF concentrations with acute forms of exercise in people living with depression, although, to date, only one study has measured the severity of depression after acute/short-term exercise. Following acute/short-term exercise, there is a temporary increase in the concentration of BDNF, which rapidly returns to baseline levels, possibly indicating a quick re-uptake by the brain, aiding its neuroplasticity functions. Overall, the evidence suggests that antidepressants also increase (or lead to an increase in) peripheral BDNF levels in patients with depression, with some evidence that there is an accompanying decrease in the severity of depression. However, the timescale of administration needed to stimulate these biochemical changes is longer than similar increases with acute exercise. Given the side effects related to antidepressants and the problems associated with adherence, along with their economic costs, it is worth considering the development of new strategies such as exercise to improve depressive symptoms, increase remission rates, and indeed shorten the time to remission in patients with depression. The message to take home is that clinicians should consider encouraging exercise in all types of depression, following a clinical assessment of the patient’s ability and willingness to exercise. They should work closely with exercise scientists to overcome the challenge of optimum exercise mode, intensity, and duration to be prescribed. Patients with low to moderate depression could benefit from exercise, leading to a reduction in antidepressant dosage and possibly the avoidance of antidepressants’ combinations that are sometimes used in severe or treatment-resistant depression types. Other patients may respond positively to such an extent that antidepressant therapy would no longer be required.

## 7 Recommendations and future directions

The results of this review revealed a significant difference between the methods used to collect the BDNF not only in post-exercise time but also in the type of fluid (serum vs. plasma), which led to different results. A plausible source of variability could be the type of ELISA test used. BDNF assessment post-intervention appears to be an increasingly important point to consider (from 10 to 60 min). We therefore recommend enhancing the standardization in measurements for future research in this field. We particularly recommend using plasma for BDNF measurements associated with short bouts of intense exercise, since the blood would be collected in tubes with anticoagulants and thus would be free of any BDNF that remained stored in the platelets after the exercise. This is also based on the most recent study by De Assis et al. (2021) who showed that metabolic stress downregulates BDNF expression but not plasma BDNF concentrations. The serum would be more relevant for measuring the effect of a long-term exercise regime on BDNF stores.

This review could be considered a wake-up call to the policymakers to start thinking if exercise could be considered as a therapy that could potentially replace medication and save a significant health-related cost for the government. This would also imply academic institutions and the governing Ministries integrate specific courses within the medical and health science curriculum enabling future medical research. One of the questions that are still to answer for instance is: should the cognitive changes be associated with other factors in depressive symptoms to warrant the exercise-related improvement? We believe that answer to such a question could be dealt with *via* medical trials comparing exercise with anti-depressants.

## Dedication

The authors mourn the death of their colleague Dr. Roger Ramsbottom (Oxford Brookes University, United Kingdom) on the 15th of February 2023. Our thoughts and sympathy go to his family *via* this article that could be one of his last published works.

## Data Availability

The original contributions presented in the study are included in the article/supplementary material further inquiries can be directed to the corresponding author.
